# Case Report: Panic attacks after tirzepatide injection in two female patients with recurrent depressive disorder

**DOI:** 10.3389/fdsfr.2026.1880796

**Published:** 2026-10-07

**Authors:** Ulrich Palm, Markus Backmund, Hubertus Himmerich

**Affiliations:** 1 P3 Klinik, Private Clinic for Psychiatry, Psychotherapy and Psychosomatics, Tutzing, Germany; 2 Department of Psychiatry and Psychotherapy, Klinikum der Universität München, Munich, Germany; 3 PIT, Praxiszentrum im Tal, Munich, Germany; 4 Department of Psychological Medicine, Centre for Research in Eating and Weight Disorders (CREW), Institute of Psychiatry, Psychology & Neuroscience, King’s College London, London, United Kingdom; 5 South London and Maudsley NHS Foundation Trust, London, United Kingdom

**Keywords:** GLP-1 receptor (GLP-1R), interaction mechanism, psychiatric disorder, side effect, weight loss

## Abstract

Tirzepatide was introduced several years ago for the management of obesity. Its side-effect profile includes gastrointestinal symptoms and hormonal shifts similar to other glucagon-like peptide-1 receptor agonists (GLP-1RAs). This class of medications is currently being investigated for its potential utility in treating depressive and anxiety disorders. While safety data generally indicate a low risk for adverse psychiatric events - such as suicidal ideation or depressive episodes - and some evidence suggests a beneficial impact on mood, anecdotal reports have linked these agents to panic attacks. However, scientific evidence for this remains limited. We present the cases of two female patients with histories of depressive disorder who experienced panic attacks following tirzepatide injections for weight loss. Both required hospitalization for the exacerbation of moderate-to-severe recurrent depressive disorder. The panic attacks occurred in close temporal proximity to the injections and resolved upon discontinuation. No clear medical aetiology was identified. Changes in pharmacokinetics by delayed gastric emptying, GABAergic deficits, an activation of the nucleus of the solitary tract (NTS) or activation of the hypothalamus-pituitary-adrenal axis are considered as potential mechanisms for panic attacks.

## Introduction

In recent years, GLP-1 receptor agonists (GLP-1RAs) have emerged as innovative and effective treatments for obesity and the management of metabolic and cardiovascular health consequences. WHO recommends long-term treatment with GLP-1RAs combined with intensive behavioural therapy to maximize and sustain benefits (Celletti et al., 2026). Furthermore, they seem to have positive effects on symptoms of depression, anxiety and binge eating and addictive behaviours (Chen et al., 2024; Tsai et al., 2022; Robert et al., 2015; Himmerich and McElroy, 2024).

At present time, tirzepatide is the only approved drug with a dual mechanism as GLP-1 and glucose-dependent insulinotropic peptide (GIP) receptor agonist, and it seems to be associated with greater weight reduction compared to the pure GLP-1 RAs, e.g., semaglutide or liraglutide (Müllertz et al., 2024). Generally, GLP-1 RAs anti-obesity medications do not only elicit gastrointestinal effects and side effects such as delayed stomach emptying (Jalleh et al., 2024) and hormonal changes, e.g., in insulin, glucagon, ghrelin, cortisol and vasopressin (Diz-Chaves et al., 2020) but also suppress appetite by affecting receptors in the central nervous system (CNS) (Beutler, 2026). As these agents have effects in the CNS and are currently being explored for specific psychiatric indications, they may also carry risks of adverse CNS effects such as anxiety, depression, and, in some instances, suicidal ideation. Rimonabant, an older anti-obesity drug, for example, was pulled from the market because it caused severe depression and suicidality (Christensen et al., 2007).

Post-marketing pharmacovigilance data of tirzepatide show a variety of medical or hormonal side effects whereas psychiatric side effects seem to be restricted to sleep disturbances and anorexia nervosa (Gu, 2026). A recent analysis of safety data showed no elevated risk for depression symptoms or suicidal ideation or behaviour in patients undergoing tirzepatide treatment compared to placebo, however these participants had no history of psychiatric illness (Wadden et al., 2026). Another study using EuraVigilance data suggested a low level of psychiatric adverse events of tirzepatide compared to other GLP1-RA. Among the rare adverse events of tirzepatide, anxiety was the most prominent, followed by depression and suicidal ideation (Tobaiqi and Elkout, 2024). Generally, tirzepatide treatment for obesity seems to be associated with a lower rate of suicidal ideation or suicide attempts compared to treatment with anti-obesity medications other than GLP1-RA, e.g., bupropion/naltrexone or orlistat (Yu et al., 2025). However, panic attacks following tirzepatide treatment are not mentioned in the scientific literature (pubmed search, search terms: tirzepatide + panic attacks, January 2020 – May 2026) albeit there are several point of view reports on social media platforms (google search, search terms: tirzepatide + panic attacks, sorted by most recent). Here, we report the occurrence of panic attacks in two female patients with history of recurrent depressive disorder, who were hospitalized upon request of their psychiatrist or psychotherapist for inpatient treatment of depression with a focus on psychotherapeutic respectively psychosomatic treatment.

## Case description

### Case 1

The 52-year-old female patient had a history of recurrent depressive disorder for 22 years. She had three episodes of depression with the last episode 9 years ago. Since then, she was treated with a stable dosage of venlafaxine 75 mg and showed no more symptoms of depression over recent years. She had no history of cardiac, pulmonary or metabolic disease and did not take any medication except for venlafaxine. Treatment with tirzepatide (Mounjaro® Kwikpen 2.5 mg) was initiated off-label upon the patient’s request for cosmetic weight loss (BMI = 27 kg/m^2^). No other injections were made at this time, and the patient was naïve to weight-loss medications. First tirzepatide injection was done around noon and in the late morning of the following day, the patient experienced panic attacks with anxiety, feelings of oppression, trepidation, dyspnea, cold shiver done the spine, nausea, and globus sensation. Nausea did not resolve after food intake but rather worsened. Panic symptoms slowly resolved towards the evening hours. The following day, the patient woke up with severe symptoms of depression, reporting depressed mood, hopelessness, despair and lack of impetus until noon. Tirzepatide was discontinued after first injection and the panic attacks subsided entirely, with no further episodes reported. Symptoms of depression continued for the following days and led to an increase of venlafaxine to 112.5 mg/d and intake of melatonin 1 mg for sleep disturbances. Two weeks after injection, the patient was hospitalized due to worsening of depression symptoms, without suicidal ideations, and was treated with an increased dosage of venlafaxine 150 mg/d and zopiclone 3.75 mg/d for insomnia. Upon admission, the Beck Depression Inventory (BDI) showed a sum score of 26 points, indicating a moderate-severe depression. Diagnostics revealed no medical reason for panic attacks. ECG was normal and laboratory works showed normal blood count, blood glucose, liver and pancreatic enzymes, kidney function parameters, and thyroid hormones. Panic attacks did not occur during inpatient treatment. Inpatient treatment consisted in daily individual and group psychotherapy. The patient could be discharged after 4 weeks of inpatient treatment with a remission of depression symptoms (BDI sum score = 8). Further outpatient treatment was carried on by her psychiatrist and psychotherapist.

### Case 2

The 49-year-old female patient had a history of recurrent depressive disorder since several years, with a first psychosomatic inpatient treatment 14 years ago for depression and anxiety and a second psychiatric inpatient treatment 3 years ago for the same symptoms. Since then, she was in a stable condition and was continuously treated with quetiapine ER 250 mg/d which was established during the former inpatient treatment for insomnia however there were no bipolar or psychotic symptoms or suicidal ideation in medical history. Two months before the first injection of tirzepatide, she experienced a worsening of depressive symptoms with unstable mood which was treated with promethazine 25–50 mg following her needs. Feelings of panic were rarely occurring but no distinct panic attacks. Suicidal ideations were absent upon admission and symptoms revealed an anxiety disorder rather than a psychotic disorder with somatic delusions. She had a history of obesity (BMI = 44 kg/m^2^) and hypertension and was continuously treated with amlodipine 10 mg/d, valsartane 160 mg/d and hydrochlorothiazide 12.5 mg/d. Following her wish for weight reduction, tirzepatide (Mounjaro® Kwikpen 2.5 mg) was begun simultaneously with a low-calorie diet. No other injections were made at this time, and the patient was naïve to weight-loss medications. Two days later, she experienced panic attacks beginning in the morning; she subsequently skipped breakfast due to nausea. However, the nausea did not resolve after food intake during day. Over the following days, she was suffering from continuous feelings of panic, feelings of oppression and tremor without intervals. Symptoms reached their peak on the fourth day and then steadily diminished. A second injection of Mounjaro® Kwikpen 2.5 mg was administered 1 week after first injection. The patient was hospitalized for moderate-severe depression (BDI sum score = 27, upon admission) on the same day of the second injection. Again, 2 days later she experienced a severe panic attack, followed by continuous feelings of anxiety and panic. Sporadic panic attacks persisted over the following days, with the final episode occurring 6 days after the second injection. Tirzepatide was discontinued after the second injection. Diagnostics revealed no medical reason for the panic attacks. ECG was normal, and laboratory works showed normal blood glucose, liver and pancreatic enzymes, kidney function parameters, and thyroid hormones, whereas there was an increase of total cholesterol and a microcytic erythrocytosis. Sertraline was initiated and titrated to a daily dose of 200 mg; quetiapine ER 250 mg and antihypertensives were continued. Inpatient treatment over 17 weeks, with an intensive psychotherapeutic regimen with daily individual and group psychotherapy sessions, led to a relief of depression symptoms, only one short episode of panic for a few seconds was reported during the latter weeks. BDI at discharge was at 10 points. Further outpatient treatment was carried on by her psychiatrist and psychotherapist.

The patient noted that the panic attacks associated with tirzepatide differed qualitatively from her previous experiences. While her prior anxiety was characterized by a gradual escalation and remained manageable, the episodes during tirzepatide treatment were described as sudden-onset, paroxysmal, and overwhelming.

## Discussion

GLP-1RAs have been reported to reduce weight in patients with preexisting psychiatric condition, albeit there is no study focusing on tirzepatide (Müller Alves et al., 2025). The reported cases indicate a chronological connection between tirzepatide injection and the occurrence or worsening of panic attacks, albeit a causal relationship is missing. In this context, assessment of causality for Case 1 reveals a score of 2 points on the Naranjo Scale, pointing to a possible adverse drug reaction, and the WHO-UMC causality assessment indicates ‘possible’. For Case 2, a score of 4 points on the Naranjo Scale as well as the WHO-UMC indicate a ‘possible’ causal relationship. Although the rechallenge of symptoms in Case 2 is more affirmative and led to a higher score in the Naranjo Scale, rigour of explanation in both cases is weakened by the potential overlap of genuine psychiatric symptoms, i.e., anxiety. A relevant limitation in this context is the lack of specific assessment of anxiety symptoms by questionnaires. Especially in Case 2, several influencing factors could have biased the attribution of panic attacks to the tirzepatide injection, i.e., the worsening of anxiety and depression before the first injection, the concomitant beginning of a low-calorie diet that could have provoked nausea and shakiness, and the hospitalisation on the same day of second injection as a stressful event. On the other hand, panic attacks were again provoked in same intensity and course by the second injection which might point to a distinct mechanism beyond psychic cause. There are several hypotheses that might explain potential mechanisms of action, but final conclusions cannot be drawn from these two case reports.

Nausea, a common side effect of GLP-1RAs (Müller Alves et al., 2025), could be attributed to tirzepatide or interpreted as a symptom of the patient’s panic attacks. The lack of resolution following food intake suggests that the nausea was not secondary to hypoglycaemia. A potential explanation could be found in the GABAergic deficit hypothesis in depression and anxiety disorders, leading to loss of prefrontal control and increased amygdala action. There is some evidence that GIP activates the inhibitory role of GABAergic neurons in rodent models (Liskiewicz et al., 2023) and reduces nausea (Borner et al., 2024). A structural or functional deficit in GABAergic neurons may impair GIP signalling, intensifying the nausea provoked by GLP-1RAs. This physiological distress could then manifest as, or trigger, overwhelming states of panic.

GLP-1RAs activate the nucleus of the solitary tract (NTS) and the area postrema. These areas are closely linked to the autonomic nervous system (Diz-Chaves et al., 2020). Overstimulation can mimic the physical sensations of a panic attack, such as rapid heart rate (tachycardia) and shortness of breath (Zoccal et al., 2014).

GLP-1 RAs can directly activate the hypothalamus-pituitary-adrenal (HPA) axis and increase the release of ACTH and cortisol (Holt and Trapp, 2016). A sudden spike in these stress hormones can trigger a physiological state of high alert that the brain interprets as panic. However, no cortisol/ACTH measurements were obtained from patients during panic attacks and this hypothesis remains elusive.

Furthermore, concomitant medication, or in Case 2, onset of a low-calorie diet, could have influenced the occurrence of panic attacks. As mentioned in the introduction, GLP-1RAs delay gastric emptying and the resorption of the patient’s psychotropic medication could have been delayed, leading to changes in bioavailability of venlafaxine, quetiapine, and promethazine. As delayed gastric emptying can alter the absorption kinetics of concurrently administered oral drugs, this could plausibly contribute to mood and anxiety symptom fluctuations independent of any direct CNS effect of tirzepatide.

Overall, these potential mechanisms need to be underpinned by further research with larger samples.

Although GLP1-RAs, i.e., tirzepatide, are deemed safe in general population (Wadden et al., 2026), there is a need for distinct assessment of risks in patients with preexisting psychiatric disorders to avoid worsening of psychiatric symptoms, although psychiatric disorders are frequently connected to obesity due to psychopharmacologic treatment, loss of impetus for physical activity, and poor and high-calorie nutrition (Müller Alves et al., 2025). Although efficient control of obesity is highly warranted in patients with psychiatric disorders, there is still insufficient evidence for the use of GLP1-RAs, i.e., tirzepatide, in this specific population (Müller Alves et al., 2025).

Both cases contrast with an earlier report of two patients with a history of depression and depression and anxiety respectively who experienced improvement of depression and anxiety symptoms under tirzepatide treatment (Mudd and Rado, 2025). Interestingly, psychiatric symptoms gradually improved with titration of tirzepatide to higher doses, independently from weight loss or stable psychopharmacologic treatment. Furthermore, in the second case report, rechallenge with tirzepatide led to recurrence of symptoms which supports a causal relationship. Overall, available data suggest no specific risk for depression or anxiety symptoms under tirzepatide treatment, and case reports suggest an improvement of mood and anxiety in patients with preexisting psychiatric disorder, contrasting our findings of panic attacks in two patients with preexisting depressive and, respectively, depressive and anxiety disorder.

Still, the mechanism for the occurrence of panic attacks remains unclear and further reports of this uncommon phenomenon under tirzepatide injection may elucidate the reasons.

## Data Availability

The original contributions presented in the study are included in the article/supplementary material, further inquiries can be directed to the corresponding author. Both cases were reported to the Dept. of Pharmacovigilance and Safety of Lilly Deutschland GmbH for further referral to central databases.
